# Helium-oxygen reduces the production of carbon dioxide during weaning from mechanical ventilation

**DOI:** 10.1186/1465-9921-11-117

**Published:** 2010-08-26

**Authors:** Gordon Flynn, Gerlinde Mandersloot, Marie Healy, Mark Saville, Daniel F McAuley

**Affiliations:** 1 Intensive Care Unit, Royal London Hospital, Whitechapel, London, E1 1BB, UK; 2 Respiratory Medicine Research Programme, Centre for Infection and Immunity, The Queen's University of Belfast, Grosvenor Road, BT12 6BN, Belfast, Northern Ireland; 3 Intensive Care Unit, Prince of Wales Hospital, Barker Street, Sydney, NSW, 2031, Australia

## Abstract

**Background:**

Prolonged weaning from mechanical ventilation has a major impact on ICU bed occupancy and patient outcome, and has significant cost implications.

There is evidence in patients around the period of extubation that helium-oxygen leads to a reduction in the work of breathing. Therefore breathing helium-oxygen during weaning may be a useful adjunct to facilitate weaning. We hypothesised that breathing helium-oxygen would reduce carbon dioxide production during the weaning phase of mechanical ventilation.

**Materials/patients and methods:**

We performed a prospective randomised controlled single blinded cross-over trial on 19 adult intensive care patients without significant airways disease who fulfilled criteria for weaning with CPAP. Patients were randomised to helium-oxygen and air-oxygen delivered during a 2 hour period of CPAP ventilation. Carbon dioxide production (VCO_2_) was measured using a near patient main stream infrared carbon dioxide sensor and fixed orifice pneumotachograph.

**Results:**

Compared to air-oxygen, helium-oxygen significantly decreased VCO_2 _production at the end of the 2 hour period of CPAP ventilation; there was a mean difference in CO_2 _production of 48.9 ml/min (95% CI 18.7-79.2 p = 0.003) between the groups. There were no significant differences in other respiratory and haemodynamic parameters.

**Conclusion:**

This study shows that breathing a helium-oxygen mixture during weaning reduces carbon dioxide production. This physiological study supports the need for a clinical trial of helium-oxygen mixture during the weaning phase of mechanical ventilation with duration of weaning as the primary outcome.

**Trial registration:**

ISRCTN56470948

## Introduction

Weaning from mechanical ventilation is estimated to account for up to 40% of the total duration of ventilatory support [1]. The process of weaning patients therefore has a major impact on ICU bed occupancy with significant cost implication [2]. Strategies to facilitate weaning have a major potential to reduce use of healthcare resources [3,4].

Helium is an inert gas and prolonged administration to animals has demonstrated no adverse effects [5]. Helium has a lower density and higher viscosity compared with oxygen and nitrogen. Breathing helium leads to a decreased resistance in gas flow, a change from turbulent to laminar flow patterns [6] and a reduction in the work of breathing. However a change from turbulent to laminar flow patterns is unnecessary for the reduction in the work of breathing which can occur under fully turbulent flow [7].

Helium-oxygen has been used in clinical situations where upper or lower airways obstruction or disease leads to an increased resistance to flow. Although there are many case reports of successful use of helium-oxygen in these conditions, to date no studies have conclusively demonstrated improved outcomes in these patient groups [8].

There are limited data regarding the use of helium-oxygen during weaning. Use of a helium-oxygen mixture during weaning with CPAP has been successfully used to improve respiratory distress and improve PaO_2 _after cardiovascular surgery in a small study in infants [9]. In addition, in ventilated patients with airflow obstruction, breathing helium-oxygen during a T-piece breathing trial just prior to extubation resulted in a reduction in airway resistance and consequently a decrease in work of breathing [10].

The aim of this physiological study was to determine whether breathing a helium-oxygen mixture as compared with an air-oxygen mixture during the weaning phase of mechanical ventilation would reduce carbon dioxide production in patients without significant airways obstruction.

## Materials and methods

We conducted a prospective single centre, randomised, single blinded, controlled, cross-over study in our 18 bed mixed medical-surgical ICU. Approval for the study was obtained from Research Ethics Committee and the Medicines and Health Regulatory Agency (MHRA). Eligible patients were ready for weaning to CPAP and had to meet the following inclusion criteria; the underlying cause of respiratory failure was improving, pressure support ventilation of less than 10 cmH_2_O, no continuous intravenous sedation or inotropes, FiO_2 _less than or equal to 0.4 and requiring less than 10 cmH_2_O positive end expiratory pressure. Written informed consent from the patient or assent from their next of kin was obtained.

Respiratory parameters were measured using a near patient main stream infrared carbon dioxide sensor and fixed orifice pneumotachograph connected to a respiratory profile monitor (CO_2_SMO Plus Respiratory Monitor, Novametrix Medical systems, Wallingford, CT, USA) and analysed using computer software (Analysis plus). The capnograph is barometric pressure compensated with an accuracy of +/- 2 mmHg (for 0 - 40 mmHg) and +/- 5% of the reading (for 41 - 70 mmHg). The pneumotachograph is a disposable device using differential pressure with an overall accuracy of +/-2%. This device was calibrated for the specific fraction of inspired helium and oxygen on an individual patient basis according to the manufacturer's instructions. On initialisation the device performs a zero calibration. The accuracy of the infrared carbon dioxide sensor is further verified by using a calibration device for carbon dioxide. Furthermore a previous study showed the monitoring device remained stable and accurate over a 48 hour period of continuous monitoring[11]. Alveolar minute ventilation, respiratory rate and CO_2 _production were continuously recorded by the CO_2_SMO plus monitor. Representative base line carbon dioxide production in a 70 kg male is 200 ml/min. An average of a 5 minute period of these parameters was recorded before the start of CPAP as a baseline and at 1 and 2 hours during each CPAP period with the study gases. Systolic and diastolic blood pressure and heart rate were recorded directly by means of an indwelling arterial catheter and electrocardiogram (ECG) attached to a bedside monitor. Arterial partial pressure of carbon dioxide and oxygen were obtained over a 2-hour period from arterial blood gas samples.

Patients were randomly assigned to initially breath either Heliox or air-oxygen mixtures. Patients were blinded to the gas mixture they received. Data was collected directly to a laptop computer and the researcher and study statistician who analysed the data were blinded to the gas mixtures. Following baseline measurements, patients received 2 hours of CPAP ventilation (PEEP setting remained unchanged and pressure support set to zero) with helium-oxygen or air-oxygen via an eVent ventilator (eVent Medical Inc. 81 Columbia Suite 101, Aliso Viejo, CA 92656). This ventilator was calibrated for the helium oxygen mixture on an individual patient basis according to the manufacturer's instructions. Patients were returned to their pre study ventilator settings for 2 hours, before being given the alternative gas mixture for 2 hours.

The level of CPAP support and FiO_2 _were unchanged for individual patients throughout the trial period. The study CPAP trial was defined as unsuccessful and discontinued if the patients developed two or more of the following criteria: respiratory rate > 40 breaths/min or rapid shallow breathing index (RSBI) > 105; SpO_2 _<90% or SpO_2 _decrease to >8% from the patients baseline value; HR > 140 beats/min or HR changes by >20% from the patients baseline; systolic blood pressure >200 mmHg or < 80 mmHg or systolic blood pressure changes by >20% of baseline; deterioration in conscious level, defined as a fall in GCS of >2, or if the patient became agitated/sweating/anxious.

The data were tested for normality and a paired t-test was used to test the treatment effect on within-subject differences. A priori the 2-hour time point was used as a summary measure of treatment effect. The data were expressed as means, standard deviations (SD) and 95% confidence intervals (CI). A p-value of < 0.05 was considered statistically significant.

## Results

Twenty-three patients were recruited into the study. A total of 19 completed the study protocol and their baseline characteristics are displayed in Table 1. Patients were treated with mechanical ventilation for a median 9 days (inter-quartile range, IQR, 6-12 days). The primary underlying condition was neurological in 8 patients, medical in 4 patients, polytrauma in 6 patients with 1 surgical patient. One patient was recruited with an infective exacerbation of COPD.

**Table 1:** Patient characteristics

Patient number	Age	Primary reason condition	Secondary reason condition	APACHE II	Status at unit discharge	Length of unit stay (rounded)	Length mechanical ventilation till inclusion
1	21	Status epilepticus or uncontrolled seizures		17	Alive	11	9
2	77	Pulmonary haemorrhage not defined	Thoracic or thoraco-abdominal aortic aneurysm	22	Alive	21	12
3	84	Inhalation pneumonitis (smoke or gases)		19	Alive	11	12
4	44	Pneumonia, no organism isolated	Depression	14	Alive	9	5
5	84	Haemorrhage or haematoma from pelvis, long bones or joints	Fractured ribs	23	Alive	30	12
6	65	Intracerebral haemorrhage	Secondary hydrocephalus	20	Alive	5	2
7	30	Traumatic rupture or laceration of liver	Hypovolaemic shock	16	Alive	17	13
8	68	Lung collapse or atelectasis	Lung abscess	16	Alive	23	6
9	46	Primary (diffuse) brain injury	Lumbar spine fracture or ligamentous injury	25	Alive	27	26
10	47	Primary (diffuse) brain injury	Amputation of limb	15	Alive	10	6
11	72	Primary (diffuse) brain injury	Traumatic subarachnoid haemorrhage	19	Alive	9	7
12	76	Abdominal aortic aneurysm, ruptured	Acute renal failure due to haemodynamic causes	18	Dead	12	7
13	45	Primary (diffuse) brain injury	Pneumonitis due to food and vomit	10	Alive	13	10
14	18	Traumatic myocardial perforation	Anoxic or ischaemic coma or encephalopathy	12	Alive	22	17
15	36	Tracheal trauma or perforation	Traumatic pneumothorax	19	Alive	20	12
16	28	Traumatic subdural haemorrhage	Focal brain injury	9	Alive	10	8
17	58	Chronic obstructive pulmonary disease with acute exacerbation, unspecified		15	Alive	3	1
18	79	Traumatic subdural haemorrhage		28	Alive	6	4
19	67	Pneumonia, no organism isolated	Pleural effusion	24	Alive	12	9
Mean	53.3			17.3		13.9	9.2
SD	21.7			5.0		7.6	5.7
Median							9

**IQR**							6-12

Four patients did not have evaluable data and were not included in the analysis. One patient became anxious when commenced on CPAP and withdrew consent (helium-oxygen), in 2 patients the respiratory rates exceeded the protocol within 15 minutes of commencing CPAP and were returned to their pre-study ventilatory support (1 helium-oxygen, 1 air-oxygen) and 1 patient was randomised but developed epileptic seizures just prior to starting CPAP and was withdrawn. Fifteen of the patients were studied on an FiO_2 _of 0.3 or less, three patients were on an FiO_2 _0.35 and one patient on an FiO_2 _of 0.4. Nine patients received helium-oxygen mixture first compared to ten receiving air-oxygen first.

**Figure 1:**
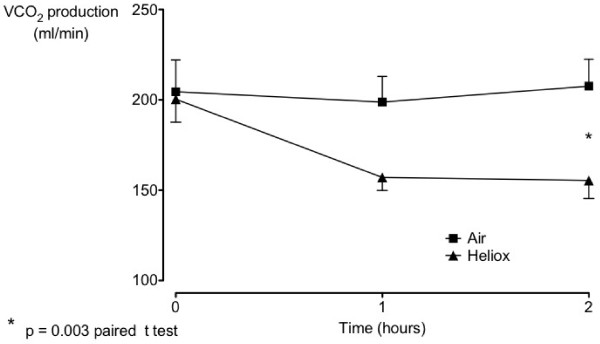
**Carbon dioxide production over time in hours for heliox and air**.

Compared to air-oxygen, helium-oxygen significantly decreased VCO_2 _production at the end of the 2 hour period of CPAP ventilation (Figure 1) There was a mean difference in CO_2 _production of 48.9 ml/min (95% CI 18.7-79.2 p = 0.003) between the groups. There were no significant differences between baseline and 2 hours CPAP with air-oxygen and helium-oxygen in all other respiratory and heamodynamic parameters measured (Table 2).

**Table 2:** Respiratory and haemodynamic parameters during the study period

	Baseline	Helium/oxygen after 2 hours CPAP	Baseline	Air/oxygen after 2 hours CPAP	Statistical significance
**RR, breaths/min**	24 +/- 7	25 +/- 5	26 +/- 6	25 +/- 6	NS
**PaCO2, kPa**	5.2 +/- 1.0	5.2 +/- 1.0	5.4 +/- 1.1	5.4 +/- 1.2	NS
**PaO2, kPa**	11.3 +/- 2.1	11.2 +/- 1.8	12.7 +/- 2.3	11.7 +/- 2.4	NS
**Minute volume**	10.2 +/- 2.8	10.8 +/- 2.5	10.6 +/- 2.3	10.2 +/- 2.4	NS
**HR, beats/min**	89 +/- 14	89 +/- 13	88 +/- 14	91 +/- 14	NS
**SBP, mmHg**	128 +/- 27	126 +/- 23	126 +/- 23	130 +/- 27	NS
**DBP, mmHg**	63 +/- 8	62 +/- 10	63 +/- 11	65 +/- 12	NS
**Temperature**	37 +/- 1	37 +/- 1	37+/- 1.5	37 +/- 1	NS

Definitions of abbreviations; RR = respiratory rate; HR = heart rate; SBP = systolic blood pressure; DBP = diastolic blood pressure; NS = non significant.

Values are means +/- standard deviation

## Discussion

Our study showed a significant reduction in CO_2 _production in patients without significant airways disease. This supports the need for a definitive clinical study of Heliox in weaning from mechanical ventilation to be undertaken. We were surprised by the 19% reduction in CO_2 _production seen while breathing helium oxygen although this is in keeping with a 21% reduction in work of breathing shown by Diehl et al in their study [10]

Weaning from mechanical ventilation has a major impact on ICU bed occupancy and patient outcome, and has significant cost implications. Strategies to facilitate weaning have a major potential to improve patient outcome and reduce the use of healthcare resources. We demonstrate in this physiological study that patients weaning from mechanical ventilation show a significant reduction in carbon dioxide production when breathing a helium-oxygen mixture. We found that all other respiratory and cardiovascular parameters measured showed no significant changes from baseline values.

In our study we used CO_2 _production as a surrogate for the work of breathing. Studies have confirmed that inspiratory muscular work of breathing is proportional to the exhaled volume of CO_2 _per minute after allowing a period of time for stabilisation of CO_2 _[12-14]. Our findings are consistent with previous studies using helium-oxygen in intubated patients with COPD during controlled ventilation and on pressure support ventilation during the weaning phase of ventilation [15,16]. These studies have shown a reduction in total, resistive and elastic work of breathing with helium-oxygen mixtures. In spontaneously breathing patients with COPD during a T-piece trial there was a reduction in work of breathing from 1.4 to 1.1 J/L in 13 patients with COPD and a reduction in intrinsic positive end expiratory pressure PEEPi [10]. Change in flow from turbulent to transitional or laminar by the use of the less dense helium is thought to be a major reason for improvement in gas flow. However, a study by Papamoschou, demonstrated that helium-oxygen does not need to be laminar to improve flow and benefits exist even if flow remains turbulent [7]. In a study in 18 patients without COPD studied immediately post-extubation, helium-oxygen given for 15 minutes reduced inspiratory effort as measured by transdiaphragmatic pressure changes. A significant subjective improvement in respiratory comfort was also observed. This benefit reversed when patients were returned to air-oxygen [17]. However as patients were already weaned to the point of extubation, no conclusion can be drawn as to whether helium-oxygen improved the weaning phase. A further small study of helium use in infants post-cardiac surgery, during weaning, showed a reduction in CO_2 _production and an increase in PaO_2 _reflecting a reduction in work of breathing [9]. Our current study extends these previous data to a group of general adult intensive care unit patients without significant airways disease during the weaning phase of mechanical ventilation. While this physiological study has demonstrated a beneficial but transient effect on CO_2 _production with the short-term use of a helium mixture, future studies designed to investigate the effect on duration of weaning would require longer term use of helium mixture.

It is worth noting that helium can interfere with the function of ventilators and in particular, flow measurement devices. It is therefore important that clinicians are aware of the effects helium can have on the equipment they use, and equipment must be compatible with, and calibrated for, use with helium [18].

This study has several limitations. The aim of this physiological study was to measure CO_2 _production in patients without documented obstructive airways disease. It is not possible to exclude that a proportion of the patients had unrecognised small airways obstruction. The study is limited by the small number of patients and one patient had a documented history of COPD. Importantly when this patient is removed from the analysis the beneficial effect of helium-oxygen is still significant. In addition, we used CO_2 _production as a surrogate for work of breathing. Carbon Dioxide production is one of the indirect calorimetric methods of measuring metabolic rate. Factors other than work of breathing that increase metabolic rate will likely increase CO_2 _production. No changes to the physical workload of our patients were made during the study period. Furthermore there was no difference in other measured respiratory and haemodynamic parameters or temperature as shown in Table 2. This indirectly indicates that the change in CO2 production is likely to indirectly reflect work of breathing. Measurements of trans-oesophageal pressures or pressure-volume loop would have been useful to more directly assess work of breathing but unfortunately these were not available.

In conclusion, our study demonstrated a significant reduction in CO_2 _production, as a surrogate measure of work of breathing, in adult patients during the weaning phase of ventilation breathing a helium-oxygen mixture. This provides support for a clinical study powered for duration of weaning as the primary outcome to be undertaken.

## Abbreviations

CO_2_: carbon dioxide; COPD: chronic obstructive pulmonary disease; CPAP: continuous positives airway pressure; DBP: diastolic blood pressure; FiO_2_: fraction inspired oxygen; HR: heart rate; RR: respiratory rate; NS: non significant; PEEPi: positive end expiratory pressure intrinsic; SBP: systolic blood pressure; RSBI: rapid shallow breathing index.

## Competing interests

The authors declare they have no competing interests. The Royal London Hospital received an unrestricted £10,000 grant from BOC to support this study which was used to purchase the CO2MO monitor. Two ventilators were loaned by BOC to the hospital for the duration of the study.

## Authors' contributions

GF participated in the study design and set up, recruitment of patients, data collection and manuscript writing. GM, MH, MS participated in patient recruitment, data collection, review of the manuscript. DM participated in study design, coordination and set up and review of the manuscript 'All authors read and approved the final manuscript.'
